# Influence of Mesenchymal Stem Cells Transplantation on Regeneration Activity of Cirrhotic Liver

**DOI:** 10.5005/jp-journals-10018-1107

**Published:** 2014-07-28

**Authors:** Agayev Rauf Maksud, Popandopulo Andrey Gennadiyevich, Jafarli Rasim Elkhan

**Affiliations:** 1 Department of Surgical Disease, Azerbaijan Medical University, Baku, Azerbaijan; 2 Donetsk Institute of Emergency and Reconstructive Surgery, International Cell Culturing Center, Donetsk, Ukraine

**Keywords:** Liver cirrhosis, Portal hypertension, Stem cells, Treatment.

## Abstract

The efficacy of mesenchymal stem cells transplantation on regeneration activity of cirrhotic liver was evaluated in an animal model of cirrhotic liver. Autologous stem cells were obtained from bone marrow. Transplantation was performed 1 week after surgery by introducing stem cells into the liver. Immunohistochemical staining (proliferative activity, myofibroblast activation and sinusoidal capillarization) was evaluated to assess the efficacy of transplantation. It was found that, 2 months after stem cell transplantation, the severity of perisinusoidal fibrosis, inflammation of the liver and the number of myofibroblasts were reduced. Stem cell transplantation may be one of the new treatment modalities for cirrhotic liver.

**How to cite this article:** Maksud AR, Gennadiyevich PA, Elkhan JR. Influence of Mesenchymal Stem Cells Transplantation on Regeneration Activity of Cirrhotic Liver. Euroasian J Hepato-Gastroenterol 2014;4(2):83-86.

## INTRODUCTION

Liver cirrhosis (LC) is a chronic progressive disease accompanied by fibrotic and regenerative restructuring of the parenchyma of the organ, hepatic and cell failure and gradual development of portal hypertension.^1^ Despite the compromised function of the liver cells, hepatocytes are capable of regeneration. However, their mass deaths as well as their reduction under the influence of various damaging factors of regenerative potential of the liver lead to gradual change of the structure of the later by its replacement by fibrous tissues.^2,3^ At present, there are many means of stimulation of the regenerative process of cirrhotic liver.^4^ However, short-term outcome has usually been recorded. It has been reported that the average length of life of patients at compensated stage of the LC is 10 years; however, it is only around 2 years in decompensated stage.^3^ At present, relatively curative treatment of LC is liver transplantation.^1^ However, the limited numbers of donor organs, severe course of the diseases as well as material and technical limitation reasons tremendously limited the opportunities of liver transplantation.

In this context, cell therapy is considered as an alternative approach to organ transplantation.^4^ In fact, stem cell (SC) transplantation may be such a maneuver.^5^ The main source of SC in the body is the bone marrow capable of generating precursor cells for a large number of body tissues.^6,7^ In animal experiments and in the clinics, it has been shown that liver regeneration can be stimulated by autologous SC transplantation.^5,6^ This study was conducted to assess the effectiveness of combined transplantation of mesenchymal SC in regeneration activity of the cirrhotic liver.

## MATERIALS AND METHODS

Studies have been performed in International Cell Cul-turing Center under Donetsk Institute of Emergency and Reconstructive Surgery and Research Center of Azerbaijan Medical University.

About 49 male albino rats (Wistar) weighing 150 to 180 gm (aged 3 month) were used for the experiments. The animal experiments were performed in accordance with the regulations of the European convention on protection of vertebrate animals used for experimental and other scientific purposes. The LC was induced by subcutaneous administration of CCl_4_ (Component reactive Co, Donetsk, Ukraine) at the rate of 0.3 ml/100 g body weight of the animal as a 50% oil solution, twice a week for 12 weeks. The animals were managed with free access to food and water. The LC formation was determined by biochemical (serum ALT, ACT, LDH and albumin) and morphological (fibrosis with the formation of connective tissue septa and false lobules, degeneration and necrosis of hepatocytes) criteria.

The autologous bone marrow SC was obtained from femoral bone.^8^ The bone marrow aspirate was diluted with Hanks’ solution (Biolot, Saint Petersburg, Russia) in a ratio of 1:2/5. The Histopaque 1.077 density gradient (Sigma, St. Louis, MO, USA) was suspended in 50 ml tubes. The diluted bone marrow was carefully layered on the gradient. Then, the tube was centrifuged at room temperature at 1800 to 2000 rpm/min for 30 to 40 minutes. The interphase cells were collected into the 15 ml centrifuge tube with little Hanks’ solution, and the suspension was resuspended. The tube was centrifuged at 800 to 1,000 rpm/min for 8 to 10 minutes. The additional deposit was decanted; the deposit was resuspended in Hank’s solution and the centrifugation process was repeated twice.^9^ Then, the deposit was mixed with DMEM/F12 (Sigma, St Louis, MO, USA), 20% ETS Biolot (Biolot, Saint Petersburg, Russia) and mitogens. Thereafter, the suspension was cultured on 75 cm^2^ plastic flasks (Nucleon, Vienna, VA, USA) in a carbon dioxide (CO_2_) incubator at 37°C for 3 days. The non-adherent cells were recovered and media was changed once in every 2 days. The SC obtained were cultured *in vitro* in IMDM with 10% ETS (Sigma), 2 mM L-glutamine and 10-4 M 2-mercaptoethanol.^10^

### Stem Cell Transplantation

To make comparative evaluation of SC transplantation, the animals were divided into two groups: group I included 24 rats who had received transplantation of SC through portal vein and the animals of group II (25 rats) received the autologous SC through the common hepatic artery.

Before transplantation, the animals were anesthetized with ketamine at the dose of 90 mg/kg and xylazine at a dose of 90 mg/kg animal weight. Administration of autologous SC to the common hepatic artery or portal vein was performed by laparotomy through injection of 1 ml cell suspension (2.0 × 10^6^ per 100 gm animal weight).

## HISTOMORPHOLOGICAL STUDY

We performed a liver biopsy, before and at the 8th week after the transplantation under ultrasound guidance. To carry out a light microscopy review, the material was fixed in 10% neutral formalin for 24 hours. Then, it was washed in running water for 2 to 3 hours, dehydrated in alcohol and embedded in paraffin by standard techniques. Paraffin sections were stained with hematoxylin and eosin for the evaluation of the morphology of the cells, with Sudan-III for the determination of lipids and by Van Gieson for the visualization of the fibrous process at the light-optical level. To assess severity of extents of liver damages, histological activity index was evaluated using ‘Knodell index’.^11^

To evaluate the effectiveness of SC administration, we also used immunohistochemical evaluation to study the proliferative activity of liver cells from the presence of expression of proliferating cell nuclear antigen (PCNA). Alpha smooth muscle actin (a-SMA) was also evaluated in liver specimens. Sinusoidal capillarization (by CD34 expression) was also evaluated.

## RESULTS AND DISCUSSION

**Figs 1A to D:**
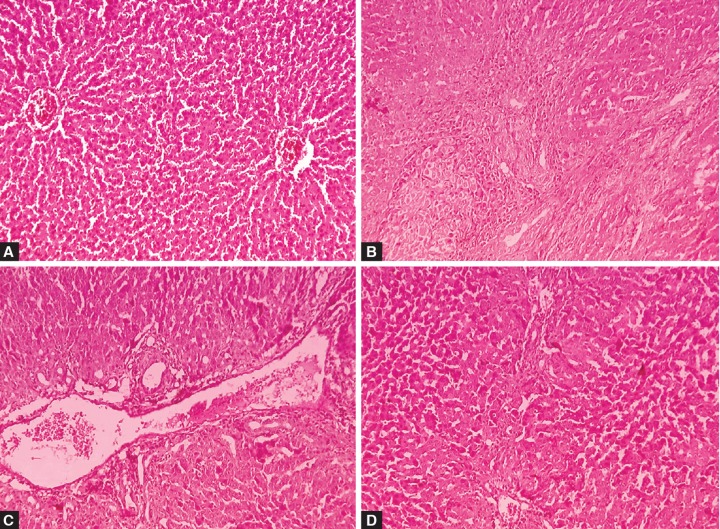
Histological section of the rat liver is in normal condition before induction of cirrhosis: (A) The morphology of liver after induction of liver cirrhosis, (B and C) the histopathology of the liver, 8 weeks after administration of stem cells and (D) magnification: 250x

The structures of liver before and after inducing LC are shown in Figures 1A to D. Liver of LC rat showed growth of the connective tissue, massive round cell infiltration of the parenchyma, sign of polymorphism and necrosis. Occasionally binucleated cells were found. Around the altered hepatocytes, accumulation of neutrophils and lymphocytes were observed. In the majority of cells, decay of nuclei was observed (Fig. 1B). Periportal and intralobular necrosis of hepatocytes were also marked in the liver of LC rat. Portal and periportal tracts with fibrosis separate the liver tissue into false lobules, bile capillaries with swollen endothelium, and lymphocytic infiltration of periportal tracts (Fig. 1C).

Histomorphological studies conducted 8 weeks after the transplantation of SC into liver showed a decrease in the intensity of the inflammatory necrotic process. However, features suggestive of necrosis of hepatocytes and subcapsular regeneration foci were determined. These may have indicate the beginning of regenerative processes in the organ. Despite the fact that false lobules persisted, binucleated hepatocytes and hepatocytes with clear cytoplasm and enlarged nuclei containing chroma-tin were detected at this point (Fig. 1D). Also, a decrease in inflammatory and necrotic processes was seen. A change in vascular component was found; sinusoids were not collapsed, and a moderate expansion of the portal and central veins was found. In the liver parenchyma, diffusely scattered binuclear hepatocytes and hepatocytes with enlarged nuclei were revealed.

**Figs 2A and B:**
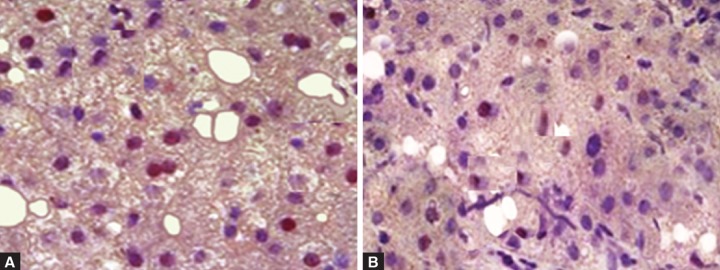
Proliferative activity of hepatocytes: (A) Before transplantation, (B) 8 weeks after transplantation. Staining with antibodies to proliferating cell nuclear antigen (PCNA) (nuclei of proliferating hepatocytes in red color) (magnification: 400x)

To assess the effectiveness of transplantation of auto-logous SC, we analyzed liver biopsy specimens before and at 8th week after transplantation for signals of PCNA, α-SMA and CD34. High proliferative activity of LC liver was seen by PCNA staining. After transplantation of SC, the proliferative activity was reduced to almost 10% of total hepatocytes (Figs 2A and B).

**Figs 3A and B:**
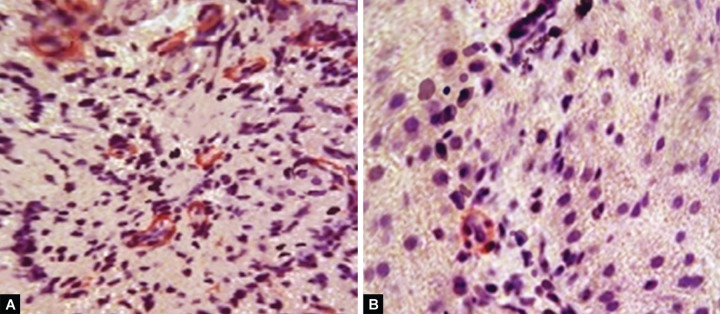
Myofibroblast activation: (A) Before transplantation (intense), (B) 2 months after transplantation (sporadic cells). Staining with α-SMA antibodies (immunohistochemical reaction product in red color) (magnification: 400x)

Significant changes were found after the trans-plantation of autologous SC in the endothelium of the sinusoids. Before the transplantation, all sinusoidal capillarization (endothelial cells expressing CD34) was massive. About 2 months after the transplantation, only sporadic CD34 endothelial cells were detected in sinusoids (Figs 3A and B).

Immunohistochemical analysis also showed positive dynamics in the expression of α-SMA. Before the transplantation, presence of myofibroblasts in connective-tissue septa (Fig. 3A) showing progression of fibrosis was detected in all animals. About 8 weeks after the transplantation, the number of myofibroblasts significantly reduced and α-SMA cells were found sporadically. They mainly localized in smooth muscle cells of blood vessels (Fig. 3B).

Given that myofibroblasts are related to progression of fibrosis, reduction of their numbers is an indicator of restoration of the normal structure of sinusoidal capillaries.

## CONCLUSION

It seems that combined transplantation of autologous SC in cirrhotic liver may be an effective method for reduction of fibrosis and induction of regeneration.
